# Can serum M30 levels be utilized as an activation marker in patients with ulcerative colitis?

**DOI:** 10.1590/1806-9282.20240704

**Published:** 2024-09-13

**Authors:** Omer Burcak Binicier, Sevil Ozer Sarı, Zehra Betul Pakoz, Banu Isbilen Basok

**Affiliations:** 1 University of Health Sciences Turkey, Izmir Faculty of Medicine, Department of Gastroenterology – İzmir, Turkey.; 2 Katip Celebi University, Ataturk Training and Research Hospital, Department of Gastroenterology – İzmir, Turkey.; 3 University of Health Sciences Turkey, Izmir Faculty of Medicine, Department of Medical Biochemistry – İzmir, Turkey.

**Keywords:** Ulcerative colitis, M30, Apoptosis, Inflammation, Cytokeratin-18

## Abstract

**OBJECTIVE:**

Ascertainment of disease activation is an important component of therapeutic decisions in ulcerative colitis patients and may present certain clinical challenges. The objective of this study was to determine serum levels of the M30 fragment of cytokeratin 18 and its utility as an activation marker in patients with ulcerative colitis, who are known to have increased apoptosis.

**METHODS:**

A total of 60 ulcerative colitis (30 active and 30 remission) patients aged over 18 years and 29 healthy individuals as controls were included in the study. M30, C-reactive protein, and mean platelet volume were evaluated in all participants and compared between ulcerative colitis patients and controls, as well as between those with active disease or remission.

**RESULTS:**

Although ulcerative colitis patients with active disease had higher M30 levels than those in remission, the difference was not statistically significant (p=0.085). The mean M30 levels tended to increase with increasing extent of involvement, although the differences were not significant (p=0.065). The comparison of C-reactive protein and mean platelet volume according to the site of involvement, however, showed significant differences (p=0.02 and 0.004, respectively). M30 did not show significant correlations with C-reactive protein, mean platelet volume, and Mayo Score (p=0.0834, 0.768, and 0.401, respectively).

**CONCLUSIONS:**

Our results suggest that, in contrast to C-reactive protein and mean platelet volume, M30 levels do not have a significant role as an activation marker in ulcerative colitis patients. Thus, we believe that M30 may not represent an appropriate marker to be used for this purpose.

## INTRODUCTION

Ulcerative colitis (UC) is an inflammatory bowel condition that affects the colonic mucosa and is clinically characterized by activations/remissions during the course of the disease^1^. It may lead to proctitis, left-sided colitis, or extensive colonic involvement. Although genetic and environmental factors have been implicated in its etiology, the exact cause remains unknown^2,3^. An increased number of T lymphocytes in the colonic mucosa represents the fundamental histologic finding, and apoptosis occurring in association with impaired T cell functions plays an important role in its pathogenesis^4^. T lymphocytes increase the expression of Fas-ligands, and increased apoptosis is observed in colonic cells with Fas expression. At the same time, increased Fas-ligation results in the activation and migration of inflammatory cells, leading to augmented apoptotic injury in colonic epithelial cells as an important pathogenetic mechanism^5^.

Ascertainment of disease activation is an important component of therapeutic decisions in UC patients and may present certain clinical challenges, including the invasive nature of colonoscopy, radiation exposure, and the inability of current activation markers to arrive at an accurate differential diagnosis^6,7^. Therefore, the identification of non-invasive and practical methods that will allow detecting UC activation represents an interesting research area^8,9^.

Cytokeratins are an epithelial-specific subgroup of intermediate filament proteins that are mainly involved in protecting cells from apoptosis and necrosis^10^. Increased serum levels of cytokeratins are a marker for apoptosis^11^. Until now, the M30 fragment of cytokeratin 18 has been found to be associated with disease severity and prognosis in a number of different conditions, including hepatocellular carcinoma, gastric carcinoma, non-alcoholic fatty liver disease, and hepatitis B^12-15^.

The objective of this study was to determine serum levels of the M30 fragment of cytokeratin 18 and its utility as an activation marker in patients with UC, who are known to have increased apoptosis.

## METHODS

Patient selection: A total of 60 UC patients aged over 18 years treated with a diagnosis of UC in our unit between December 2018 and May 2019 were included. Also, 29 healthy ­individuals served as controls. The diagnosis was based on clinical, ­endoscopic, and histopathological findings.

Study design: Patients were categorized into two groups, namely, those who had active disease based on clinical and endoscopic findings and those who were in remission. The disease activation was assessed using the Mayo Score, which rated stool frequency, rectal bleeding, mucosal appearance, and Physician's Global Assessment on a 0–3-point scale, yielding a total Mayo Score between 0 and 12^16^. The site of involvement was grouped into three, namely, proctitis, left colitis, and extensive colitis. Also recorded were the ­medications used for UC treatment. M30, C-reactive protein (CRP), and mean platelet volume (MPV) were evaluated in all ­participants and compared between UC patients and controls, as well as between those with active disease or ­remission. The ­correlations between M30 and CRP, MPV, and Mayo Score were analyzed.

Exclusion criteria: Age under 18 years, history of intestinal ­surgery, presence of active infection, coexistent hepatic and/or renal failure, presence of chronic disorders, pregnancy, and lactation.

Laboratory methods: After an overnight fast, venous blood ­samples were collected into plain blood collection tubes (BD Vacutainer^®^ SST II Advance Tube, 5 mL, 13×100 mm, United States). Serum ­samples were separated from cellular fragments by centrifugation for 10 min at 1,500 *g* within 1 h after blood sampling. Serum samples were aliquoted and stored at -80°C until further analysis.

Serum M30 concentrations were determined by a ­commercial kit employing a quantitative sandwich enzyme ­immunoassay technique (Human Cytokeratin 18-M30, SinoGeneClon Biotech Co. Ltd., Hangzhou, China).

## STATISTICAL ANALYSIS

Statistical analysis of the study was done using SPSS 25.0 (IBM Statistical Package for Social Sciences software version 25). Continuous variables were expressed as a ­mean±standard ­deviation and categorical variables as a percentage. The chi-square test was used to compare categorical values, and the Mann-Whitney U test was used to compare continuous ­variables between groups. Receiver operating characteristic (ROC) ­analysis was performed to calculate the cutoff values. An area under curve (AUC), positive predictive value (PPV), and negative predictive value (NPV) were obtained. Correlations between M30, CRP, and Mayo Score were determined using Spearmen's rho test. A p-value less than 0.05 was considered statistically significant.

Ethical considerations: The study protocol was approved by the local ethics committee (approval no: 2019/14-37). All patients and controls provided written informed consent for study participation.

## RESULTS

Of the 60 UC (30 active, 30 remission) patients and 29 healthy controls, 47 (52.8%) were male and 42 (47.2%) were female. Women comprised 40 and 62.1% of the patient and control groups, respectively. There was a significant gender difference between patient and control subjects (p=0.005). The mean age was 45.1±13.8 years (range: 18–71 years) in patients diagnosed with UC patients and 46.0±15.4 years (range: 18–81 years) among controls, with no significant difference (p=0.366). Patients with active disease or in remission were ­comparable in terms of the site of involvement, disease duration, and treatments received (p=0.148, 0.450, and 0.196, respectively).

Ulcerative colitis patients and controls had comparable M30 levels (p=0.132). Although UC patients with active ­disease had higher M30 levels than those in remission, the difference was not statistically significant (p=0.085). However, MPV levels were significantly different between patients with active disease or in remission (p=0.01). Also, significant differences in CRP were found both between UC patients and controls and between UC patients who had active disease and were in remission (p=0.03, and<0.001, respectively). Table 1 shows a comparison of UC patients with active disease or in remission with respect to laboratory parameters.

**Table 1:** Comparison of ulcerative colitis patients with active disease or in remission versus control subjects.

	Active (n=30)	Remission (n=30)	Controls (n=29)	p
M30, pg/mL (mean±SD)	605.34±794.63	307.56±116.85	360.53±96.47	0.132* 0.085**
CRP, mg/dL (mean±SD)	4.99±4.79	0.45±0.44	0.33±0.4	0.03* <0.001**
MPV, fL (mean±SD)	8.19±1.23	8.8±0.95	8.54±0.75	0.01* 0.806**

SD: standard deviation; CRP: C-reactive protein; MPV: mean platelet volume.

* Comparison for active disease-remission.

** Comparison between UC patients and controls.

Also, M30, CRP, and MPV differences according to the site of involvement were also examined. The mean M30 ­levels tended to increase with increasing extent of involvement, although the differences were not significant (p=0.065). The comparison of CRP and MPV according to the site of involvement, however, showed significant differences (p=0.02 and 0.004, respectively). Table 2 summarizes M30, CRP, and MPV according to the site of involvement.

**Table 2:** M30, C-reactive protein, and mean platelet volume levels according to the site of disease involvement.

	Rectum (n=12)	Left colitis (n=25)	Extensive colitis (n=23)	p
M30, pg/mL (mean±SD)	272.7±79.9	475.4±490.9	531.9±787.4	0.065
CRP, mg/dL (mean±SD)	0.78±0.94	1.50±1.94	5.06±5.53	0.02
MPV (fL) (mean±SD)	8.31±0.76	9.1±1.12	7.98±1.03	0.004

CRP: C-reactive protein; MPV: mean platelet volume; SD: standard deviation.

Correlations between M30, CRP, MPV, and Mayo Score were evaluated. Accordingly, M30 did not show significant correlations with CRP, MPV, and Mayo Score (p=0.0834, 0.768, and 0.401, respectively). However, there was a significant correlation between MPV and CRP (p=0.036). Similarly, Mayo Score and CRP were significantly correlated (p<0.001).

**Figure 1:**
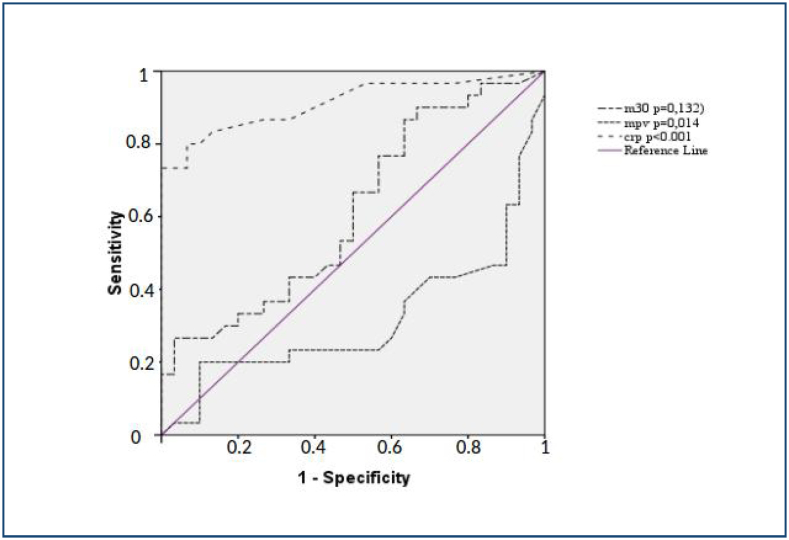
Mean and confidence interval for scores in question 4, according to observer group.

A ROC curve analysis was carried out to examine the ­efficiency of M30, CRP, and MPV in assessing the disease ­activity in UC patients. This analysis showed that while MPV and CRP had significant efficiency, M30 had no statistically ­significant role in showing the disease activity (p=0.014, ≤0.001, and =0.132, respectively) (Figure 1).

## DISCUSSION

Non-invasive methods for ascertaining UC activation have been an interesting research area. Obviously, such non-­invasive ­methods should ideally be practical and reliable, as well as ­represent a viable alternative for endoscopic procedures. Currently, CRP and calprotectin are commonly used for this purpose^17^. On the contrary, CRP is frequently elevated in many other acute conditions, such as infections, limiting its utility in UC activation assessments^18^. Reduced MPV is another parameter used to evaluate UC activation although similar to CRP, MPV ­levels may also be diminished in certain infectious, rheumatoid, or cardiac conditions^19,20^.

Cytokeratin 18 is synthesized in epithelial cells and is involved in many cellular processes such as apoptosis, mitosis, and stress response. Serum levels of the M30 fraction of cytokeratin 18 have been investigated in hepatic, lung, kidney, and many other cancer types, with some studies reporting significant results^21^. M30, which is a substrate for caspase 3, is a marker of apoptosis. M30 serum levels are increased due to the loss of membrane integrity following cell death and can be detected using the ELISA method^16,22^. These observations suggested that M30 may also represent an appropriate marker for assessing the disease activity in UC since apoptosis plays a major role in the pathogenesis of UC. Apoptosis is increased mainly due to increased activity of TNF, interleukins, and interferons. Also, inflammation destroys the integrity of the mucosal barrier in patients with acute UC activation^23^.

Drug therapy targeted at reducing apoptosis in colonic cells is associated with mucosal healing in UC patients^24^. Furthermore, endoscopic activation and microscopic ­inflammation are strongly linked with epithelial apoptosis^25^. Thus, an assumption was made that M30 could assist in differentiating active UC patients and patients in remission. In contrast with CRP levels, UC patients and control subjects did not differ significantly in M30 levels in the current study. Again, in contrast with CRP and MPV, UC patients with active disease or in remission were not significantly different in terms of M30 levels as well. We also failed to find any correlations between M30, CRP, MPV, and Mayo Score. A ROC curve analysis suggested a significant association between UC disease activity and MPV and CRP, while no such associations were observed for M30.

In a 2012 study by Aktaş et al., patients with active UC had significantly higher M30 levels as compared to those in remission, supporting the role of increased apoptotic activity in subjects with active disease^26^. Although mean M30 levels were highest in patients with active disease and lowest in controls, the differences were not statistically significant in the current study. Again, patients in remission and with active disease did not differ significantly in this respect.

Another hypothesis that was tested was that increased length of disease involvement would be associated with increasing levels of M30. In a study by Ueyama et al., increased FasL expression was found in patients with active extensive colitis and in those with left-sided involvement, while those with rectal involvement only had no such increase, suggesting that patients with proctitis may have less apoptotic activity^5^. Thus, we assumed that M30 levels would be higher with increasing disease extent, due to increased inflammation and apoptosis. Despite a trend toward increased M30 levels with increasing extent of the disease as compared to those with rectal involvement only, these differences did not reach statistical significance. Conversely, a significant association of CRP and MPV with the site of involvement was found.

The most important limitation of our study is the small sample size. Studies with larger sample sizes would certainly provide more meaningful results. Also, there was a significant gender difference between patients and controls. Treatments administered for UC may have an effect on the measured levels of the parameters investigated. Again, no histological assessment of the inflammation and apoptosis was performed. Another limitation was that we did not correlate M30 levels with fecal calprotectin. Fecal calprotectin cannot be used routinely in our country yet since only a few laboratories can perform the test.

## CONCLUSION

Our results suggest that, in contrast to CRP and MPV, M30 levels do not have a significant role as an activation marker in UC patients. Thus, we believe that M30 may not represent an appropriate marker to be used for this purpose. However, based on certain trends observed in this study as well as the published data, we recommend further studies with larger sample sizes.
